# Early Markers of Ongoing Action-Effect Learning

**DOI:** 10.3389/fpsyg.2012.00522

**Published:** 2012-11-27

**Authors:** Hannes Ruge, Ruth M. Krebs, Uta Wolfensteller

**Affiliations:** ^1^Department of Psychology, Neuroimaging Center and Institute of General Psychology, Biopsychology, and Methods of Psychology, Technische Universitaet DresdenDresden, Germany; ^2^Department of Experimental Psychology, Ghent UniversityGhent, Belgium

**Keywords:** instrumental learning, goal-directed action, differential outcomes, anticipation, instruction

## Abstract

Acquiring knowledge about the relationship between stimulus conditions, one’s own actions, and the resulting consequences or effects, is one prerequisite for intentional action. Previous studies have shown that such contextualized associations between actions and their effects (S-R-E associations) can be picked up very quickly. The present study examined how such weakly practiced associations might affect overt behavior during the process of initial learning and during subsequent retrieval, and how these two measures are inter-related. We examined incidental (S-)R-E learning in the context of trial-and-error S-R learning and in the context of instruction-based S-R learning. Furthermore, as a control condition, common outcome (CO) learning blocks were included in which all responses produced one common sound effect, hence precluding differential (S-)R-E learning. Post-learning retrieval of R-E associations was tested by re-using previously produced sound effects as novel imperative stimuli combined with actions that were either compatible or incompatible with the previously encountered R-E mapping. The central result was that the size of the compatibility effect could be predicted by the size of relative response slowing during ongoing learning in the preceding acquisition phase, both in trial-and-error learning and in instruction-based learning. Importantly, this correlation was absent for the CO control condition, precluding accounts based on unspecific factors. Instead, the results suggest that differential outcomes are “actively” integrated into action planning and that this takes additional planning time. We speculate that this might be especially true for weakly practiced (S-)R-E associations before an initial goal-directed action mode transitions into a more stimulus-based action mode.

## Introduction

Common sense as well as an extensive body of literature suggests that higher organisms can learn to associate perceived changes in the environment with their own actions and use this acquired knowledge to actively pursue these environmental effects (E) by choosing the right action in a given context. In the simplest choice situation successful behavior requires response R1 under stimulus context S1 but response R2 under stimulus context S2. In other words, an organism needs to be able to discriminate between “good” and “bad” outcomes (O) of action (e.g., under S1: R1 returns good outcome; R2 returns bad outcome). This discrimination can be based on some form of performance feedback or based on instruction (Doll et al., 2009; Ramamoorthy and Verguts, 2012; Wolfensteller and Ruge, 2012). There is ample evidence that such outcome discrimination is indeed an integral part of the associational structure controlling action selection (Urcuioli, 2005; Balleine and Ostlund, 2007; de Wit and Dickinson, 2009; Nattkemper et al., 2010). That is, different from the classical Thorndikian view, performance feedback or “reinforcement” does not only serve the imprinting of stimulus-response (S-R) associations, but is in fact becoming part of a triple S-R-O or S-R-E association (Silvetti and Verguts, 2012). This is the associational basis of goal-directed action, enabling an agent to select an action based on anticipating the likely outcome this action would entail under a certain stimulus context. To disentangle S-R imprinting and S-R-O learning, the use of “differential outcomes” (DO) has been adopted in a wide range of different paradigms, including the term-defining “*DO paradigm*” (Trapold and Overmier, 1972), the *selective outcome devaluation paradigm* (e.g., Colwill and Rescorla, 1985), the *selective outcome priming paradigm* (e.g., Elsner and Hommel, 2001; Ziessler et al., 2004), and the *natural outcome compatibility paradigm* (e.g., Hommel, 1993; Kunde, 2001). In all these paradigms different actions do not only entail a common positive/negative feedback but additionally each action entails a unique outcome. This can be different types of rewards (e.g., sucrose liquid, food pellet, etc.) as in the outcome devaluation paradigm or different types of non-incentive perceptual events (sounds, colors, etc.) as in the selective outcome priming paradigm. Each paradigm has shown unique effects after the introduction of DO which support the notion of truly goal-directed action representations. In the DO paradigm, the trial-and-error learning rate of novel S-R mappings is higher under DO conditions as compared to common outcome (CO) conditions, especially early during learning (i.e., when error rates are still high). In the outcome devaluation paradigm, actions that have been learned to produce a certain outcome are less frequently chosen under extinction after this outcome has been selectively devaluated. In the selective effect priming paradigm, presentation of DO as response primes has shown to selectively activate those actions that have produced these effects in a preceding acquisition phase. Finally, in the natural outcome compatibility paradigm actions that produced the naturally expected effects (e.g., forcefully pushing a button leads to loud tone) were faster as compared to actions that produced the naturally incompatible effect.

Notably, these paradigms fall into one of two research traditions which share a common perspective on goal-directed action in terms of the DO rationale, but differ decisively in certain procedural aspects. One important difference is the amount of practice. Paradigms following the ideomotor learning tradition (i.e., selective effect priming and natural effect compatibility) typically investigate the impact of (S-)R-E associations after quite extended R-E acquisition periods typically amounting to more than 100 pairings of a response with its effect (amounting to a virtually infinite number of pairings for natural R-E mappings used in the compatibility paradigm). By contrast, paradigms following the instrumental learning tradition typically examine the impact of S-R-E associations early *during* (DO paradigm) or *after* (outcome devaluation) a rather limited number of S-R-E pairings well below 100 R-E pairings. Considering evidence mainly from brain research that too much practice diminishes the influence of goal (i.e., effect or outcome) representations while habitual control based on S-R associations alone becomes increasingly dominant (Killcross and Coutureau, 2003; Atallah et al., 2004; Yin and Knowlton, 2006; Seger and Spiering, 2011), it seems likely that ideomotor paradigms might measure different aspects of goal-directed action than instrumental paradigms. One speculation is that *early during learning* the anticipation of a specific outcome might affect response selection in two different ways concurrently. First, outcome anticipation might activate associations between actions and rewards (i.e., retrieving the information that one but not another response will yield reward or success in a given stimulus context). Second, outcome anticipation might directly activate the associated response, yet without any reference to its incentive value. By contrast after *extended practice*, only this latter “non-incentive” path might still be impacting behavior. This distinction might explain why extended practice reduces the impact of outcome devaluation (reference to incentive properties gets lost) while at the same time action effects are still able to prime the associated response directly via bi-directional R-E associations (for a recent review, see Wolfensteller and Ruge, 2012).

Before this background, we recently started conducting experiments within the ideomotor framework using the selective effect priming procedure, but different from previous studies we employed a comparably short R-E learning phase that is more similar to instrumental learning protocols in terms of the number of repeated R-E pairings (Wolfensteller and Ruge, 2011). In these initial experiments we found the typical effect priming results when re-using effect stimuli as response primes that were consistently produced by specific actions in a preceding acquisition phase. Specifically, test phase performance was impaired when the currently required response was incompatible vs. compatible with the response that had produced the current effect prime in the preceding learning phase. This clearly indicates that R-E associations were formed after very few (8–12) repeated pairings of R and E and, importantly, that these associations can be detected with the “passive” effect priming procedure. Hence, this demonstrates that the typical ideomotor mechanisms seem to operate even after very limited practice.

In the present study we aimed to link more directly performance measures associated with *initial ongoing* (S-)R-E learning with post-learning measures of R-E associational strength. Similar to instrumental learning protocols we implemented both, short DO learning blocks as well as short CO learning blocks. In both conditions, subjects had to learn novel S-R mappings by trial-and-error. The comparison of performance learning curves between DO and CO conditions thus allowed us to determine one index reflecting the “active” integration of goal information *during the initial acquisition* of S-R mappings. Additionally, similar to ideomotor learning protocols, after DO learning blocks were completed, an effect priming procedure was employed that allowed us to obtain a second, independent index of the strength of bi-directional R-E/R-O associations acquired beforehand. Based on these two behavioral indices, we aimed to determine how ongoing DO learning might be related to the test phase R-E compatibility effect. The rationale was that the size of the R-E compatibility effect serves as an index of R-E associational strength that can hence be used to determine the extent to which learning-related changes in performance might reflect the (increasing) incorporation of *anticipated* outcome information in action planning processes. This is particularly important in order to determine whether R-E associations are actually integrated during action planning when the natural order of events is preserved (i.e., S, then R, then E) as is the case during the initial learning phase in the present study. As of yet, evidence for active effect integration under the natural event order rests on studies involving *well practiced* associations during conditions of *R-E competition* (Kunde, 2001; Kunde et al., 2004). However, *weakly practiced* (S-)R-E associations have only been shown to “passively” impact action planning within the selective effect priming paradigm, that is, when the natural order of events is reversed (i.e., previous E, then S, then R; Wolfensteller and Ruge, 2011).

## Experiment 1

### Materials and methods

#### Subjects

Fifty subjects participated in this experiment and received monetary compensation or course credit. Data from one subject were lost due to logging errors. Hence, data analysis was based on a sample of 49 subjects (20 male, mean age24).

#### Design

**Figure 1:**
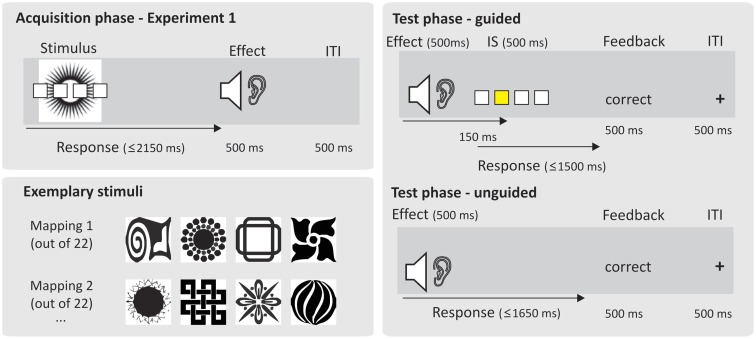
**Overview of the experimental procedures employed in Exp. 1**. ITI refers to inter-trial interval. For Exp. 2A and Exp. 2B, this procedure was used in a modified form.

The experiment comprised 22 experimental blocks, including 11 CO blocks and 11 DO blocks. CO and DO blocks were randomly intermixed. Each block comprised a learning phase in which subjects had to learn by trial-and-error novel 4:4 stimulus-response mappings. Stimuli were four abstract visual patterns (see Figure 1 for an example) that were different for each block (i.e., 88 different visual stimuli overall). The 22 sets of four stimuli were compiled such that the four stimuli within each set were easily discriminable. The sequence of the 22 sets was randomized across subjects. Hence, across subjects, each set of stimuli was equally likely to be assigned to the CO or DO condition. Responses were to be made with the left middle finger, the left index finger, the right index finger, and the right middle finger mapped to the keys “D,” “F,” “K,” and “L” on a standard “QWERTZ” keyboard. In CO blocks a correct response was followed by a CO (natural sound). This CO sound (e.g., a ring tone, a dog’s bark, a laugh, squeaking breaks, etc.) was different for each block (i.e., 11 different CO sounds overall). In DO blocks instead, correct responses were consistently followed by one of four different outcomes (again natural sounds). The four sounds were different for each block (i.e., 44 different DO sounds overall). As for the visual stimuli, we created 22 sets of four different sounds that were arranged to be easily discriminable (i.e., 88 different sounds overall). The sequence of the 22 sets was randomized across subjects. For the 11 DO blocks all four sounds were used whereas for the 11 CO blocks only one out of the four sounds was selected. Hence, across subjects, each set of sounds was equally likely to be assigned to the CO or DO condition. For both CO and DO conditions, a trial-and-error learning block was terminated when each response had been performed correctly eight times (not necessarily in a row). Alternatively, learning was terminated when a total of 70 learning trials were exceeded. For nine subjects this happened once for a single learning block each and for two subjects this happened twice. However, terminal error rates in each block were below 10% in all these cases. Since this indicates that the S-R mappings had been learned well, data were not excluded from the analysis.

A learning trial started with the presentation of the visual stimulus (S) in the center of the screen which remained on screen until response execution or time out after 2150 ms. The sound effect was presented immediately after correct response execution for 500 ms. In case of erroneous responses, error feedback was displayed for 500 ms in the center of the screen (German for “error” or “too slow”). The next trial started after a constant inter-trial interval of 500 ms.

Specific for the DO condition, the initial trial-and-error learning phase was directly followed by a “test” phase in which subjects were now required to respond to the previous effect sounds with the same set of four responses used during the acquisition phase, which could be either compatible or incompatible to the response that produced a specific effect during the preceding acquisition phase (see Elsner and Hommel, 2001, Exp. 2). The rationale is that the strength of bi-directional R-E associations acquired during the learning phase should be expressed in relatively impaired performance in incompatible vs. compatible test trials due to non-intentional response priming. Compatible and incompatible trials were randomly intermixed. The 4:4 sound-response mappings were explicitly instructed during an initial instruction phase spanning the first three presentations of each sound. During this initial “guided phase,” the correct response was instructed via yellow squares appearing on the screen and localized spatially compatible with the four responses (see Figure 1). The initial three presentations of each of the four sounds were pseudo-randomly intermixed during the first 12 instruction trials such that each sound was exactly three times correctly responded to. Erroneous trials were immediately repeated. A guided trial started with the presentation of a fixation cross in the center of the screen for 500 ms, followed by of one of the previous effects sounds which lasted for approximately 500 ms. The instructional stimulus (IS) was presented 150 ms after sound onset until response execution or until time out after 1500 ms. Response execution was immediately followed by accuracy feedback presented centrally on the screen for 650 ms (German words for “correct,” “error,” or “too slow”). For the next 24 “unguided” trials six presentations of each sound were again pseudo-randomly mixed such that each sound was correctly responded to exactly six times. The timing of trial events was exactly the same as in the guided phase. The only difference was that the IS was not displayed, hence there was a response deadline of 1650 ms relative to sound onset (instead of 1500 ms relative to IS onset).

During both the guided and the unguided test phase, two sounds were paired with the response that had produced that sound during the preceding learning phase (R-E compatible condition). The two other sounds were paired with responses that had produced a different sound in the preceding learning phase (R-E incompatible condition). Compatible and Incompatible trials were randomly intermixed. The assignment of fingers and response hands was counterbalanced across test blocks such that compatible and incompatible responses were always only partly assigned to different hands. For instance, when the index finger of one hand was assigned to the compatible condition the middle finger of the same hand was assigned to the incompatible condition. The four different assignment schemes conforming to this rule were pseudo-randomly assigned to subjects and test blocks such that each assignment scheme was realized approximately equally often. The effect of R-E compatibility was computed for the 24 test trials following the instruction phase. Note that in the CO condition, in which all responses were associated with one common effect, no test phase was administered.

#### Analysis

In the initial trial-and-error acquisition phase, the progress of learning was analyzed with regard to error rates and response times as a function of correctly implemented distinct stimulus repetitions (SRep) one through eight. For instance, SRep level 1 comprised the performance data from the first correct implementation of each of the four different stimuli occurring in a given learning block. Response times were based on the arithmetic mean across distinct stimuli and learning blocks. Analogously, error rates were expressed in terms of the percentage of errors committed. These learning curves were separately computed for CO and DO learning blocks. To assess the effect of CO vs. DO on learning performance we run two separate repeated measures ANOVAs under SPSS (version 18), one for error rates and one for response times, each with the two within-subject factors SRep and OUTCOME. To account for possible non-sphericity in the 8-level SRep factor, significance tests were based on the multivariate analysis output.

In DO blocks, the test phase R-E compatibility effect was computed both for mean response times and mean error rates based on the 24 unguided test trials. Statistical significance was assessed via paired *t*-tests.

The central analysis targeted the correlation between learning phase performance dynamics and test phase compatibility effect in DO blocks. The rationale was that the size of the R-E compatibility effect serves as an index of R-E associational strength that can hence be used to determine the extent to which learning-related changes in performance might reflect the (increasing) incorporation of outcome information in action planning processes. To this end, we computed a series of across subjects correlations between “ongoing learning” as derived from all DO and CO blocks, respectively and the mean R-E compatibility effect derived from all test phases following DO blocks. To capture “ongoing learning” we used performance at SRrep level 2 as reference for performance at the six subsequent SRep levels 3 through 8. That is, for each subject we obtained a series of mean difference values (i.e., SRep2 – Srep3, SRep2 – SRep4, etc.). Note that we decided against SRep 1 as reference even though it might appear especially well suited due to its neutral status with respect to R-E associational strength (equaling zero). However, general considerations and the actual data pattern observed at SRep level 1 suggest potential problems with this approach. Generally, it should be kept in mind that the correlational analysis aims to identify learning-related performance indices related to the active integration of learned (S-)R-E associations into action planning by exploiting inter-subject variability specifically linked to that process. In this respect, SRep1 is not an ideal reference as it comprises especially strong “nuisance” variance components related to stimulus novelty or related to confusion due to the intermixing of CO and DO blocks. Hence, irrespective of CO or DO condition, the variability caused by such nuisance variables might mask a systematic, but comparably weak variability component induced by the process of interest. Second, even though associations between stimuli and DO are by definition non-existing at SRep1, we observed a highly significant response slowing for DO trials relative to CO trials at SRep1. This effect strongly indicates the presence of DO-related processes at SRep1 that cannot be due to the active integration of learned (S-)R-E associations in action planning.

We computed the correlations between DO learning performance slope (i.e., SRep2 – Srep3, SRep2 – SRep4, etc.) and test phase compatibility. Analogously, we computed the correlations between CO learning performance slope and test phase compatibility. Although compatibility was not defined for CO blocks (and hence no test phase was implemented), this analysis was nevertheless important as a control procedure to exclude the possibility that correlations observed for the DO condition might reflect unspecific effects. For instance, participants with weak trial-and-error learning performance (indicated by small differences between the SRep2 reference and subsequent SRep levels) might also be those that are more strongly affected by R-E compatibility. In such a case we would expect a negative correlation between learning performance slope and RE compatibility for both DO and CO learning performance slope although there is no direct link in terms of R-E associations. In contrast, a specific link between DO learning and RE compatibility directly related to the acquisition of R-E associations would be expressed in a correlation exclusively for DO learning but not for the CO learning condition. To explicitly test whether these correlations were significantly different between DO and CO, we correlated the compatibility effect with the difference between DO-related learning performance slope and CO-related learning performance slope [e.g., (SRep2 – SRep3)_DO_ – (SRep2 – SRep3)_CO_]. In other words, we tested whether the compatibility effect would be associated with DO vs. CO differences in performance *slope*. In addition, we also tested whether the compatibility effect would be associated with the difference between DO and CO conditions in terms of the respective absolute performance *levels* (e.g., SRep3_DO_ – SRep3_CO_).

### Results

#### Learning performance

**Figure 2:**
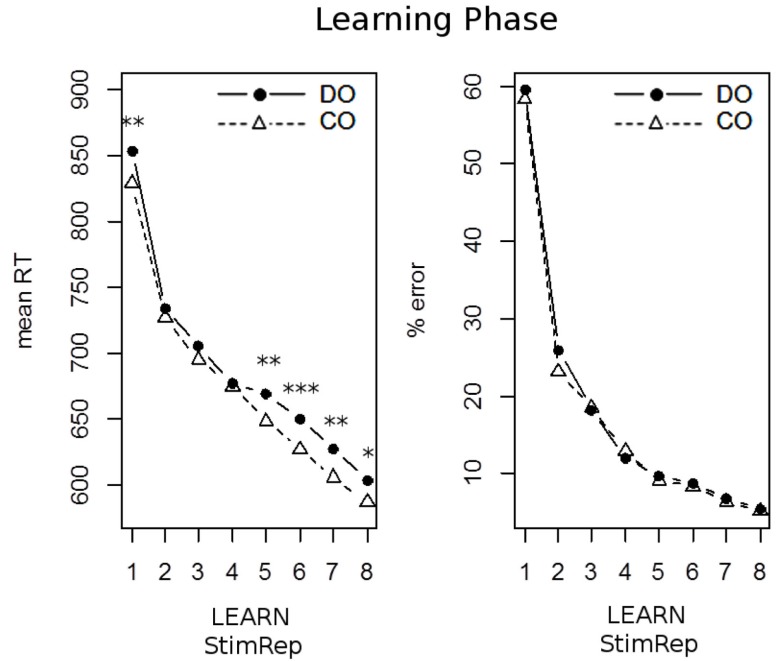
**Performance across the initial learning phase of Exp. 1 for mean response times (left panel) and mean% errors (right panel)**. DO denotes the Differential Outcome condition, CO denotes the Common Outcome condition. Learning is expressed in terms of correctly implemented stimulus repetitions (StimRep). Asterisks denote significant differences between DO and CO (*p < 0.05; **p < 0.01; ***p < 0.001).

A summary of learning performance is depicted in Figure 2. There was a sharp decline across SRep 1 to SRep 8 in error rates (*F*_7,42_ = 165.4; *p* < 0.001; $\eta_{p}^{2}$ = 0.965). However, this main effect was not significantly modulated by OUTCOME condition (*F*_7,42_ = 0.92; *p* = 0.501; $\eta_{p}^{2}$ = 0.133). Also, there was no main effect of OUTCOME (*F*_1,48_ = 1.7; *p* = 0.204; $\eta_{p}^{2}$ = 0.033). For mean RTs there was also a significant decline across correctly implemented SRep levels 1 through 8 (*F*_7,42_ = 64.3; *p* < 0.001; $\eta_{p}^{2}$ = 0.915). Different from error results, there was a highly significant OUTCOME main effect, indicating slower responses in the DO as compared to the CO condition (*F*_1,48_ = 11.8; *p* < 0.001; $\eta_{p}^{2}$ = 0.197). Again, the interaction between SRep and OUTCOME failed to approach significance (*F*_7,42_ = 1.6; *p* = 0.154; $\eta_{p}^{2}$ = 0.214). However, numerically the OUTCOME response slowing effect seemed to follow a 3-phasic pattern (see Figure 2), being strong in the beginning (SRep1), then reduced (SRep 2 through 4), and increasing again (SRep 5 through 8). In an exploratory *post hoc* polynomial contrast analysis, this 3-phasic pattern was confirmed statistically by a significant third order (cubical) interaction (*F*_1,48_ = 6.0; *p* = 0.018; $\eta_{p}^{2}$ = 0.111). Furthermore, separate paired *t*-tests (two-sided) for each SRep level revealed significantly increased RT for DO vs. CO exclusively at SRep 1 (25 ms; *t*_48_ = 2.8; *p* = 0.008; η^2^ = 0.140), SRep 5 (19 ms; *t*_48_ = 2.6; *p* = 0.014; η^2^ = 0.123), SRep 6 (23 ms; *t*_48_ = 3.5; *p* = 0.001; η^2^ = 0.203), SRep 7 (21 ms; *t*_48_ = 2.8; *p* = 0.007; η^2^ = 0.140), and SRep 8 (17 ms; *t*_48_ = 2.0; *p* < 0.057; η^2^ = 0.077).

#### R-E compatibility

We found significant compatibility effects (i.e., incompatible vs. compatible in the DO condition) for both mean RTs (545.4 ms vs. 531.5 ms; *t*_48_ = 2.73; *p* = 0.009; η^2^ = 0.134) and mean error rates (12.6 vs. 9.4%; *t*_48_ = 5.33; *p* < 0.001; η^2^ = 0.372).

#### Correlations

We first analyzed correlations between R-E compatibility and ongoing learning in terms of performance *slope* (i.e., SRep2 – SRep3, Srep2 – SRep4, etc…) for all four combinations of RT and error rate in these two inter-dependent variables (i.e., RT_learn_ × RT_test_, RT_learn_ × errors_test_, errors_learn_ × RT_test_, and errors_learn_ × errors_test_). We first did this separately for both the DO and the CO learning conditions.

**Figure 3:**
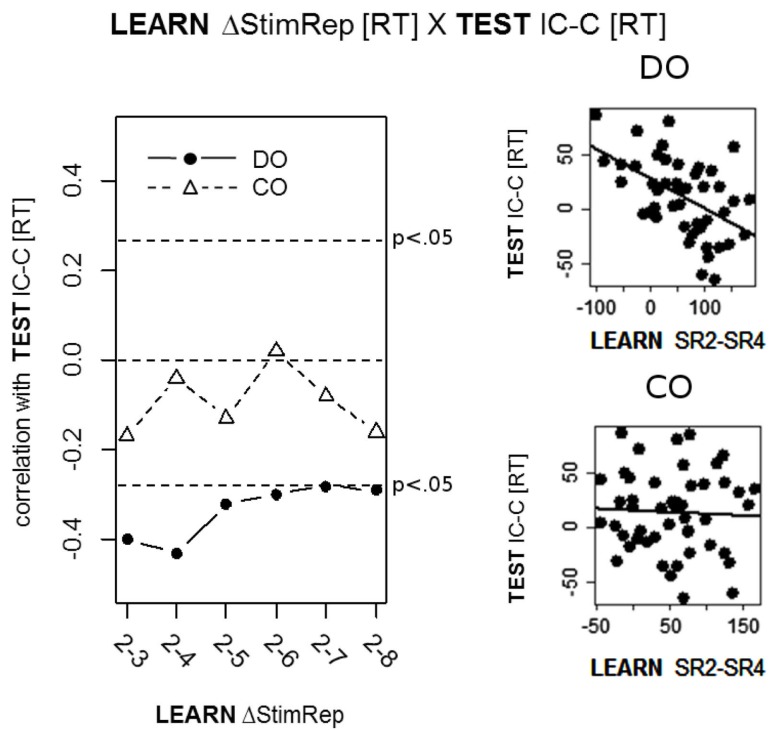
**Correlations between response times (RT) during ongoing learning and RTs during the subsequent response-effect (R-E) compatibility test in Exp. 1**. Ongoing learning is expressed in terms of the RT difference between Stimulus Repetition (StimRep) 2 and the subsequent StimRep levels (i.e., ΔStimRep). R-E compatibility is expressed in terms of the RT difference between incompatible (IC) and compatible (C) test trials.

Generally, we only found significant results for RT_learn_ × RT_test_ correlations. Specifically, we found significant *negative* correlations between R-E compatibility and learning performance slope in the DO condition for all six slope levels (all *p* < 0.05; *two-sided*)^1^. As shown in Figure 3 the correlations were strongest early during learning, peaking at SRep2-SRep4 (*r* = −0.43). Importantly, there were no significant correlations between R-E compatibility and performance slope during ongoing learning in the CO condition. These differential correlational patterns for DO and CO could be confirmed statistically for the early phase of learning by computing the correlation between the compatibility effect and the learning-related RT “difference of the difference” [e.g., (SRep2-SRep3)_DO_ – (SRep2-SRep3)_CO_]. The respective correlations were *r* = −0.36(*p* = 0.014; *two-tailed*) for SRep2-SRep3 and *r* = −0.31 (*p* = 0.031; *two-tailed*) for SRep2-SRep4.

All correlations between R-E compatibility and absolute performance level differences between DO and CO (SRep1_DO_ – SRep1_CO_, SRep2_DO_ – SRep2_CO_, etc.) revealed no significant results (all |*r*| < 0.23; all *p* > 0.122).

### Discussion

As a first important result we found that trial-and-error S-R learning under DO conditions relative to CO conditions prolonged mean response times. At the same time we did not observe the typical DO-related relative reduction in error rates early during trial-and-error learning (e.g., Mok and Overmier, 2007; Noonan et al., 2011). That we failed to replicate this latter effect on error rates is not surprising given that the learning problem was comparably easy as indicated by the sharp drop of errors from SRep1 (60%) to SRep2 (25%). Logically, DO can only start contributing to response selection from SRep2 onward as subjects need to complete SRep level 1 to know which specific DO is produced by which specific response. Given the comparably low error rate at SRep2,it seems likely that the direct S-R link is already sufficiently strong on its own, hence reducing the potential contribution of DO for selecting the correct response. Notably, mean response slowing for DO vs. CO blocks was already present at SRep 1, that is, when DO could not be known prior to response execution and could thus not directly affect response selection. Instead, SRep1 response slowing might be related to the additional effort to encode (S-)R-E associations once a subject is realizing that the present block involves DOs instead of COs. Alternatively, it might indicate increased distraction due to the higher perceptual load in the DO condition. The absence of DO-related mean response slowing in the subsequent SRep levels 2 through 4 suggests that this initial effect is rather short-lived.

Importantly, neither the RT difference between DO and CO at SRep1 nor the DO-related learning performance slope from SRep1 to SRep2 (see footnote 1) yielded significant correlations with the R-E compatibility effect as a measure of R-E associational strength. This suggests that the initial DO-related mean RT slowing effect at SRep1 has no direct relevance for the formation and usage of (S-)R-E associations. This suggests that DO-related mean response slowing at SRep level 1 might rather reflect unspecific side effects possibly related to stronger distraction from the main S-R task by the higher perceptual load imposed by DOs as compared to COs.

By contrast, when considering DO-related learning performance from SRep level 2 onward we indeed found evidence for the integration of outcome information in action planning during the learning phase. Specifically, this was indicated by strong DO-specific negative correlations between learning performance *slope* and the R-E compatibility effect. In turn, this suggests that increasing outcome integration slows down (i.e., decelerates) the overall learning-related decrease in RT. Surprisingly, the strongest negative correlations between DO-related performance *slope* and R-E compatibility effect were observed early during learning (SRep2 through SRep4) where the *mean* RT difference between DO and CO was not significant. By contrast, later during learning (SRep5 through SRep8) the negative correlations with performance slope decreased considerably while at the same time mean RTs were now significantly slower for DO as compared to CO. Similarly surprising, absolute performance *level* differences between DO and CO conditions (i.e., SRep2_DO_ – SRep2_CO_, SRep3_DO_ – SRep3_CO_, etc…) did not correlate with the R-E compatibility effect – neither early during learning (i.e., SRep2 through 4) nor late during learning (i.e., SRep5 through 8). This seems particularly contradictory for the early phase of learning where performance slope exhibited the strongest correlation with R-E compatibility. For instance, a subject who exhibits a strong DO-specific decrease in performance slope between SRep2 and SRep4 should automatically also exhibit a relative RT slowing for DO vs. CO at SRep4 as a direct consequence of the decreased slope. Hence, both measures (performance slope and performance level) should similarly correlate with R-E compatibility. Yet, only slope but not level showed the correlation.

To account for both, the dissociation between slope-related correlations and level-related correlations and between slope-related correlations and mean RT differences, we need to consider the specific nature of correlations. First, the dissociation between slope-related correlations and mean RT difference might be due to the fact that we are dealing with correlations based on inter-individual *variability* on the one hand and *mean* differences on the other hand. Hence, strong correlations are likely to emerge when there is large inter-individual variability in a behavioral marker of interest while at the same time this marker might not be strongly expressed in mean differences between conditions exactly as a consequence of this variability. Accordingly, it seems reasonable to assume that variability in the learning and/or usage of (S-)R-E associations is stronger early in learning. This might explain why *mean* RT slowing for DO vs. CO conditions is maximal later during learning (i.e., SRep5 through SRep8) when most of the subjects might have learned the underlying (S-)R-E associations to a certain extent. By contrast, earlier in learning (i.e., SRep2 through SRep4) subjects might vary strongly in the (S-)R-E learning success which might give rise to the stronger correlation with the compatibility effect. In other words, a subject who starts earlier with (S-)R-E learning (i.e., implicating a strong decrease in performance slope between SRep2 and SRep4) will have acquired stronger R-E associations by the end of the learning phase, hence giving rise to a stronger compatibility effect. At the same time, early DO-related decrease in performance slope between SRep2 and SRep4 might be present only in a relatively small proportion of subjects which might imply that overall mean RT will not be strongly increased for DO vs. CO at this stage yet.

Alternatively – or additionally – it is well conceivable that the DO-related decrease in performance slope (linked to compatibility) is not the only factor that affects response times differently for DO and CO. If this additional factor X caused a speed-up of RT for DO relative to CO, this would counteract the opposite mean RT slowing effect caused by the DO-related decrease in performance slope associated with factor Y. Hence, in sum, DO-related RT increase due to factor Y and DO-related RT decrease due to factor X might cancel out. This could explain the absence of significant mean RT differences between DO and CO for SRep2 through SRep4. Moreover, if factor X was *not* correlated with R-E compatibility [i.e., not specifically related to S-(R-E) learning/usage], this could explain why the mean RT difference between DO and CO was not correlated with compatibility. The reason is that the additional source of DO-related variability caused by factor X would overshadow the variability component caused by factor Y (i.e., the component that is associated with R-E compatibility)^2^. An additional source of “factor X” variance that might cause a mean speed-up for DO vs. CO could be related to an unspecific phasic alerting of DOs relative to COs with respect to the subsequent learning trial. Phasic alerting is known to induce a response speeding and is stronger for more salient accessory stimuli (Stahl and Rammsayer, 2005; Jepma et al., 2009). Since each of the four DOs is occurring less frequently than a single CO, its salience should be stronger and hence its phasic alerting impact on the processing of the next trial should be stronger. Clearly, this DO-related phasic alerting should be unrelated to the learning/usage of (S-)-R-E associations. Hence, this variance component should be uncorrelated with the R-E compatibility effect.

Finally, we need to discuss whether the DO-related negative correlation between performance slope and R-E compatibility indeed reflects the active integration (i.e., “usage”) of DOs in action planning processes as we had originally reasoned. Alternatively, this correlation might also be related to the learning of (S-)R-E associations itself. Maybe the most compelling scenario why (S-)R-E learning might be associated with a decrease in learning performance slope is based on the indirect influence of DO-related distraction from the main S-R task. Such distraction might increase attention toward the differential action effects which, in turn, might increase (S-)R-E strengthening. Hence, a subject who is more strongly distracted by the DOs as reflected by greater decrease in performance slope would form stronger (S-)R-E associations as reflected by a greater R-E compatibility effect. What speaks against this interpretation is that distraction should be maximal in the beginning of learning and decrease toward the end of the learning phase. Indeed, we observed a quite strong slowing effect already at SRep 1 which is likely due to unspecific distraction (see also point further above). However, this initial slowing effect did not correlate with the R-E compatibility effect, suggesting that associated initial DO-related distraction did not amplify (S-)R-E learning.

Together, we conclude that the DO-specific negative correlations between learning performance slope and R-E compatibility likely indicate the active integration or usage of newly acquired (S)R-E associations in action planning when the natural order of events is preserved (i.e., S, then, R, then E) – at least when performing in an early phase of practice as in the present study. However, it is also clear that the complex pattern of DO-related correlations and mean RT differences between DO and CO conditions suggests that learning under DO conditions involves additional unspecific processes (distraction and phasic alerting) that affect mean RT without impacting the strength of (S-)R-E associations. This latter conclusion in particular needs to be confirmed by future research that will need to disentangle the different DO-related processes that are strongly intermingled in the present study.

## Experiment 2A and 2B

To further validate and generalize the correlational results from Exp. 1, we conducted an analogous correlational analysis for two additional Exp. 2A and 2B. Different from Exp. 1 these additional experiments employed an “instruction-based” learning procedure for acquiring novel 4:4 S-R mappings instead of trial-and-error learning. Also, S-R learning took always place in a DO learning context.

### Subjects

Forty-five subjects were recruited that had not participated in Exp. 1. Twenty-five subjects participated in Exp. 2A (eight male, mean age 27) and 20 subjects participated in Exp. 2B (five male, mean age 24).

### Procedure

#### Learning phase

The instruction procedure for acquiring novel S-R mappings was structurally highly similar to the instruction procedure used for the R-E compatibility test in Exp. 1. That is, the to-be-acquired 4:4 stimulus-response mappings were explicitly instructed during an initial instruction phase spanning the first three presentations of each stimulus. Stimuli were drawn from the same set of abstract pictures as in Exp. 1 and were different for each learning block. As in the DO condition of Exp. 1 correct responses were consistently followed by one of four different natural sounds as outcomes drawn from the same set of sounds as in Exp. 1. The four sounds were different for each block. There were 20 different learning blocks, each followed by an R-E compatibility test phase.

During instructed S-R learning, the initial three presentations of each of the four stimuli were pseudo-randomly intermixed during the first 12 instruction trials such that each stimulus was correctly responded to exactly three times. During this initial “guided phase,” the correct response was instructed differently in Exp. 2A and 2B. In Exp. 2A the correct response was indicated by a yellow square appearing on the screen and localized spatially compatible with the four responses (see Figure 1). In Exp. 2B the correct response was indicated by a letter (D, F, K, or L) presented in the center of the screen. Letters were mapped to fingers according to their standard QWERTZ keyboard position (see Figure 1). Prior to the start of the experiment, subjects were told to memorize this mapping (the actual results confirmed that this was sufficiently easy for all subjects as indicated by SRrep level 1 error rates of below 20% for each subject and an overall mean SRep 1 error rate of 8%). These two instructional mappings were designed to manipulate retrieval effort, an aspect that is not further elaborated on in the present paper. Here, we simply use these two conditions for cross-validation assuming that they are sufficiently similar with regard to the processes of primary interest in the present context. A guided trial started with the presentation of the visual stimulus in the center of the screen until response execution. The IS was presented 150 ms after stimulus onset until response execution or until time out after 1500 ms. The sound effect was presented immediately after correct response execution for 500 ms. In case of erroneous responses, error feedback was displayed for 500 ms in the center of the screen (German words for “error” or “too slow”). The next trial started after a constant inter-trial interval of 500 ms.

Following the first 12 guided trials, 24 unguided trials were presented comprising six presentations of each stimulus that were again pseudo-randomly intermixed such that each stimulus was correctly responded to exactly six times. During this phase no IS was presented, yet the overall timing remained exactly the same as in the guided phase, implicating a response deadline of 1650 ms relative to stimulus onset (instead of 1500 ms relative to IS onset). Erroneous trials were immediately repeated during all phases of the experiment.

#### Test phase

The R-E compatibility test procedure used in Exp. 2A was identical to the test procedure in Exp. 1. The test procedure used in Exp. 2B was the same, except that letters were used for instruction (as in the learning phase of Exp 2B).

### Analysis

The analysis was performed analogously to Exp. 1, with two exceptions. One difference was that we used SRep level 4 instead of SRep level 2 as reference for determining the progress of learning. This was done to adjust for the fact that the first 3 SRep levels in Exp 2A and 2B were guided and hence not easily comparable to the subsequent unguided trials. Accordingly we used the first unguided SRep level 4 as reference. The second difference was that we applied a one-sided instead of a two-sided significance test for the correlational analysis according to the *a priori* hypothesis derived from Exp. 1 that the correlation should be negative. Since one might argue that Exp. 2 is not sufficiently similar to Exp. 1 to justify a one-sided test, we additionally indicate whenever significance would be missed according to the more conservative two-sided criterion. We first analyzed data collapsed across Exp. 2A and 2B. For cross-validation of the correlational results, we performed the correlational analysis separately for each sub-experiment.

### Results

#### Learning phase

**Figure 4:**
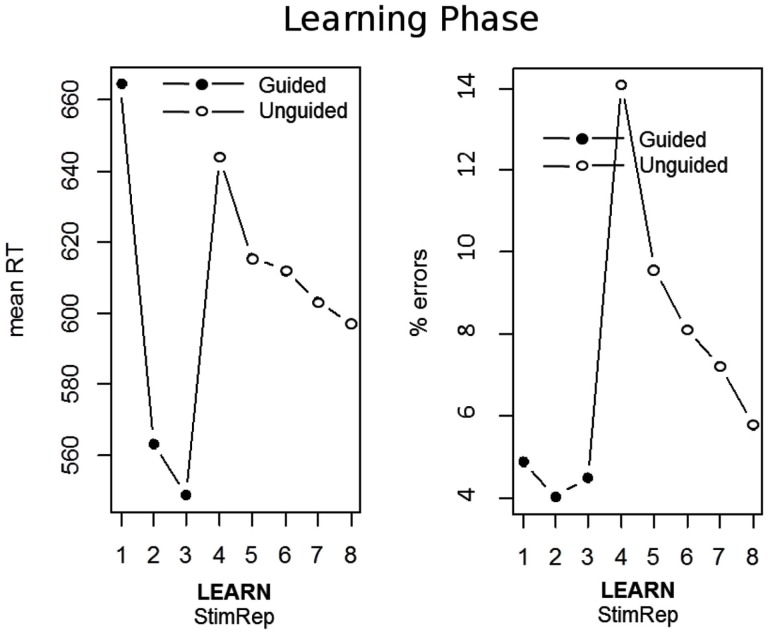
**Performance across the initial learning phase of Exp. 2A and Exp. 2B averaged together**. The left panel depicts mean response times (RT) and the right panel depicts mean% errors. Learning is expressed in terms of correctly implemented stimulus repetitions (StimRep). The first 3 stimulus repetitions were guided by an instructional stimulus (IS) whereas the remaining 5 stimulus repetitions where unguided. RTs for StimRep 1–3 are measured relative to the IS onset whereas RTs for StimRep 4–8 are measured relative to the stimulus itself.

Learning performance was analyzed separately for the guided and unguided phase using separate repeated measures ANOVAs for RTs and error rates analogous to Exp. 1. As could be expected, error rates were constantly (*F*_2,43_ = 1.4; *p* = 0.246; $\eta_{p}^{2}$ = 0.063) low during the guided phase and jumped up with the start of the unguided phase reflecting that responses had to be generated without the help of the instructional stimuli (Figure 4). Across the unguided phase error rates declined considerably (*F*_4,41_ = 18.0; *p* < 0.001; $\eta_{p}^{2}$ = 0.637). Response times declined significantly during the guided phase (*F*_2,43_ = 44.5; *p* < 0.001; $\eta_{p}^{2}$ = 0.674) as well as during the unguided phase (*F*_4,41_ = 10.8; *p* < 0.001; $\eta_{p}^{2}$ = 0.514). Note that absolute RTs were referenced to the instruction stimulus in the guided phase and to the antecedent stimulus (i.e., the abstract picture) in the unguided phase^3^.

#### R-E compatibility

As in Exp. 1, R-E compatibility (i.e., incompatible vs. compatible test trials) was determined for the unguided phase. Again replicating the results from Exp. 1, there was a significant effect for RTs (522ms vs. 511 ms; *t*_44_ = 2.7; *p* = 0.009; η^2^ = 0.142) as well as for error rates (14.8 vs. 11.3%; *t*_44_ = 6.2; *p* < 0.001; η^2^ = 0.466).

#### Correlations

**Figure 5:**
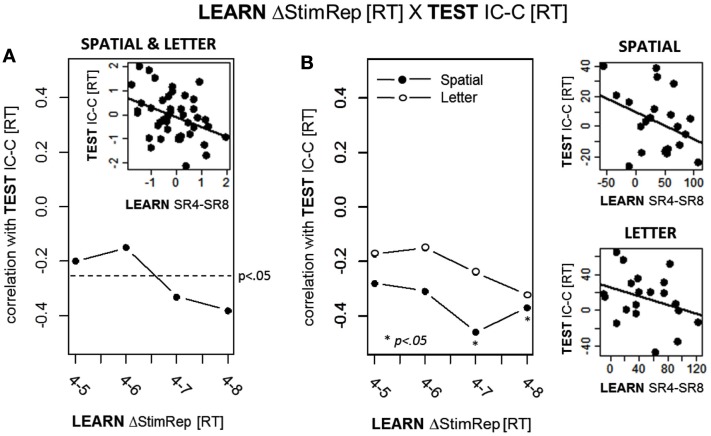
**Correlations between response times (RT) during ongoing learning and RTs during the subsequent response-effect (R-E) compatibility test in Exp. 2A and 2B**. Ongoing learning is expressed in terms of the RT difference between Stimulus Repetition (StimRep) 4 and the subsequent StimRep levels (i.e., ΔStimRep). R-E compatibility is expressed in terms of the RT difference between incompatible (IC) and compatible (C) test trials. **(A)** depicts the results of the correlational analysis across both sub-experiments. **(B)** depicts the results of the correlational analysis separately for each sub-experiment (SPATIAL and LETTER).

First, we computed the correlations between ongoing learning (i.e., SRep4 – SRep5, SRep4 – SRep6, etc.) and RE compatibility across Exp. 2A and 2B. To adjust for possible differences in the distributions of the two inter-dependent variables in each sub-experiment, we first *z*-standardized the values for each sub-experiment (mean = 0; SD = 1) before they were entered into the overall correlational analysis. We performed this analysis for all four combinations of RT and error rate in the interdependent variables (i.e., RT_learn_ × RT_test_, RT_learn_ × errors_test_, errors_learn_ × RT_test_, and errors_learn_ × errors_test_). As in Exp. 1 we found significant results only for correlations involving RTs in both inter-dependent variables. Replicating Exp. 1, the correlations between ongoing learning and RE compatibility were again all *negative*. These results are depicted in Figure 5A showing that negative correlations reached significance for SRep4 – SRep7 (*r* = −0.33; *p* = 0.016) and for SRep4 – Srep8 (*r* = −0.38; *p* = 0.006). Note that without prior experiment-wise normalization, the correlational pattern turns out to be highly similar (*r*_SRep4−Srep5_ = −0.04, *p* = 0.403; *r*_SRep4−Srep6_ = −0.13, *p* = 0.203; *r*_SRep4−Srep7_ = −0.29, *p* = 0.030; *r*_SRep4−Srep8_ = −0.36, *p* = 0.009). Figure 5B depicts the correlations based on non-standardized variables separately for each sub-experiment. Generally, both experiments yielded a similar pattern of negative correlations, reaching significance in Exp. 2A (spatial) for SRep4 – SRep7 (*r* = −0.46; *p* = 0.0135) and for SRep4 – SRep8 (*r* = −0.37; *p* = 0.044; note that this latter correlation would not reach significance with a two-sided test). However, a direct comparison of these correlations (after Fisher-*z* transformation) between Exp. 2A and 2B did not yield significant differences.

### Discussion

Generally, Exp. 2 replicated the correlation between learning-related response slowing and the R-E compatibility effect already observed in Exp. 1. However, the detailed time course of this correlation differed between experiments. Specifically, the size of the correlation *decreased* with learning in Exp. 1 whereas it *increased* in Exp. 2. While these different patterns might not be overly surprising given the procedural and analytical differences (i.e., trial-and-error vs. instructed; different reference SRep levels), some elaboration seems warranted. In particular, the diverging results might be suited to clarify whether learning-related response slowing directly indicates the strengthening of (S-)R-E associations (i.e., the process of association formation itself) or rather the “active” usage of increasingly stronger (S-)R-E associations. We propose that the results support the latter account for two reasons. First, the terminal associational strength after learning seems to be the same for Exp. 1 and Exp. 2 as suggested by the finding that the R-E compatibility effect did not differ between both experiments (tested via identical procedures). Second, the number of distinct learning trials (i.e., the number of co-occurrences for a particular S-R-E triple at each stimulus repetition level) was the same (i.e., 8) for Exp. 1 and Exp. 2 (as erroneous responses were never followed by an effect sound). Together this suggests that also the time course of associational strengthening across consecutive stimulus repetition levels can be expected to be similar in Exp. 1 and Exp. 2. Hence, the diverging time course of the correlations is unlikely to be associated with associational strengthening itself. Rather, it appears reasonable to assume that it reflects differences in the active usage of these associations. Why exactly the active usage might occur at different points in time in Exp. 1 and Exp. 2 remains unclear and requires additional experimental work. Finally, separate assessments of Exp. 2A and 2B revealed that both sub-experiments show a trend for negative correlations for later SRep levels, but this trend was numerically stronger for Exp. 2A and reached significance only for Exp. 2A. While this numerical difference between Exp. 2A and 2B could not be confirmed statistically, it still seems conceivable that the more demanding instructional S-R mapping used in Exp. 2B (i.e., letters) vs. Exp. 2A (i.e., spatial) might indeed absorb cognitive resources that could otherwise be devoted to the “active” incorporation of action effects during the learning phase. Hence, it might be worth pursuing this issue more systematically and with increased statistical power (which is clearly lacking for the between-subjects comparison of Exp. 2A and 2B).

## General Discussion

The present series of experiments aimed to establish whether, and if so, in which specific way response-contingent DO or effects might be “actively” integrated into action planning during an early phase of (S-)R-E learning (i.e., prior to considerable automatization or overlearning). We did that by investigating the relationship between performance indices of ongoing (S-)R-E learning and behavioral measures of post-learning “passive” R-E priming^4^. Exp. 1 compared S-R trial-and-error learning under DO conditions vs. CO conditions. The results suggest that DO are “actively” integrated into action planning and that this takes additional planning time as indicated by relative response slowing in terms of decreased learning performance *slope* in DO vs. CO learning blocks. This finding was replicated in Exp. 2A and 2B where novel S-R mappings were learned via instruction rather than by trial-and-error. Importantly, it seems important to emphasize that in Exp. 1 R-E compatibility was exclusively associated with a DO-specific decrease in learning performance *slope* but not with the relative DO vs. CO difference regarding absolute performance *level*. As elaborated extensively in the discussion of Exp. 1, this implies that mean RT differences between DO and CO conditions during learning might to some extent also reflect unspecific DO-related side effects. Specifically, increased perceptual load in the DO blocks might result in distraction from the primary task which might possibly result in mean response slowing for DO vs. CO blocks. Yet, this does not seem to be functionally related to the acquired strength of (S-)R-E associations^5^.Additionally, DOs might be more salient than COs which might imply stronger phasic alerting effects in the subsequent trial which might cause faster mean RTs in DO than in CO trials. Again, this potential DO-related RT speeding effect does not seem to be functionally related to the acquired strength of (S-)R-E associations.

We propose that the correlational results can in particular potentially clarify an important theoretical issue. It is entirely unclear whether newly acquired (S-)R-E associations should affect overt choice behavior in situations where the primary S-R learning task is rather easy and hence, would not decisively benefit from additional action retrieval cues in form of anticipated effects (see further below). Importantly, this question cannot simply be answered by demonstrating post-learning passive priming effects as expressed in the R-E compatibility effect. While the compatibility effect shows that R-E associations were learned, it does not tell whether these associations were already integrated in action planning during initial learning. This differentiation is not trivial as the retrieval of R-E associations triggered by direct perceptual input (i.e., the former E serving as the imperative stimulus in the test phase) does not automatically also imply that effect representations are activated through *anticipation* during the preceding learning phase (de Wit et al., 2009). By relating learning-related and test-related behavioral indices, the present study addressed and positively answered this question. Moreover, we think that it is also not trivial to show that newly acquired (S-)R-E associations affect response times negatively rather than positively. Possible theoretical implications of this aspect are discussed further below.

Next, we will critically evaluate these findings with regard to the existing literature. First we will discuss how our results relate to previous findings that also support the notion that anticipated outcomes or effects play an active role during action planning. The classical DO paradigm has demonstrated – mostly in lower animals, young children, and mentally handicapped persons – that the rate of trial-and-error S-R learning is initially higher under DO vs. CO conditions (Trapold, 1970; Mok and Overmier, 2007; Noonan et al., 2011) suggesting an active role of stimulus-based effect anticipation early in learning. The present study differs in three important aspects from this classical approach. First, the typical DO results have been obtained with incentive outcomes as compared to non-incentive outcomes used in the present study. Second, the typical DO results refer to error rates rather than response times. Notably, this also includes the few DO studies conducted in healthy adult human subjects (Mok and Overmier, 2007; Noonan et al., 2011). This exclusive focus on error rates might be related to the choice of quite challenging learning problems. While this is suited to create a slow and gradual decrease in error rates – hence increasing the potential benefit of DOs – it might at the same time imply rather noisy RT data especially in the initial learning phase where DOs have been shown to exert strongest impact on error rates. By contrast, the primary S-R learning task in the present study was comparably easy resulting in an atypically rapid decline in error rates. Not surprisingly, under these circumstances we could not detect a significant impact of DOs on error rates. Instead, DOs affected RTs. This opens a question that has not been directly addressed before, namely whether the presence of DOs should be expected to exert a positive or negative impact on RTs (see further below).

But why, in the first place, would we be interested in examining how DOs affect performance in the context of atypically easy learning problems, and hence, evaluate response times instead of error rates? The reason is that we wanted to make sure to examine the impact of DOs before any considerable automatization or overlearning of (S-)R-E associations could be expected. Therefore we restricted the number of specific S-R-E pairings to no more than eight, which is well below the number occurring for difficult learning problems. This decision was partly led by the suspicion that the typical DO studies might fail to observe significant RT effects not only in early, highly error-prone phases of learning where the accuracy-related DO effect is maximal, but also in later phases where error rates have stabilized at low asymptotes and do no longer differ between DO and CO conditions. While early in learning high RT noise levels due to high error rates might easily mask potential DO-related RT effects, the same does *not* hold for later learning phases. Hence, the suspected absence of DO-related RT effects after more extended practice (together with the typically reported absence of effects in accuracy) might in fact suggest a diminishing engagement of goal-directed control with extended practice. In the light of an extensive body on instrumental learning literature, such a conclusion is consistent with the notion that goal-directed control of action is transitioning into stimulus-based control of action already after comparably modest amounts of practice (Killcross and Coutureau, 2003; Atallah et al., 2004; Seger and Spiering, 2011). Accordingly, by strongly limiting the learning duration in the present experiments, we reasoned that the newly formed (S-)R-E associations would be actively used for action planning. Indeed, our results did confirm this expectation, as detailed above. However, it would be premature to extrapolate that the RT slowing effect would have vanished after more extended practice in the present experimental paradigm, as predicted by the instrumental learning literature. Interestingly though, Exp. 1 indeed suggests a decline of (S-)R-E usage already across the rather short learning phase as indicated by a decreasing correlation between RT slowing and RE compatibility effect. By contrast, Exp. 2 seems to suggest exactly the opposite. Hence, this issue needs to be clarified by future experiments. This seem particularly warranted in the light of results from the natural R-E compatibility paradigm (Hommel, 1993; Kunde, 2001; Kunde et al., 2004) that seem to directly contradict the hypothesis that only weakly practiced (S-)R-E associations are integrated into active action planning. Specifically, it has been shown that maximally over learned R-E associations (e.g., forceful button press – loud sound) interfere with modestly practiced R-E associations acquired within a session that are incompatible with the natural mapping (e.g., forceful button press – low sound). These and results by Ziessler and Nattkemper (Ziessler et al., 2004; Ziessler and Nattkemper, 2011) have been interpreted to reflect the active integration of *anticipated* action effects into action planning. A possible re-conciliation might be that the experienced incompatibility between natural effects and newly introduced reversed effects triggers a switch back to a goal-oriented action mode. Note that outcomes are always compatible in the classical DO paradigm, hence precluding the “forced” adoption of a goal-oriented action mode. However, this hypothesis still needs to be directly tested.

Next we discuss possible explanations for why active effect-based action planning was associated with response slowing instead of response facilitation in the present study. Intuitively and contrary to the actual results, a speed-up of response times under DO vs. CO conditions might seem more plausible. Such an expectation might be implied by the idea that the anticipation of a specific DO provides just another valid retrieval cue for the currently required response in addition to the antecedent stimulus cue. Hence, the correct response code is activated “twice” which implies that the response threshold is reached earlier than under CO conditions where this additional retrieval cue is absent. This would be consistent with results from the passive effect priming procedure suggesting that RTs are shorter for compatible effect primes as compared to neutral primes (Ziessler et al., 2004). Clearly, however, at least for weakly practiced (S-)R-E associations, this scenario is not supported by the present results. Instead, the observed DO-related response slowing might indicate that effect-based action selection should be conceptualized as an additional time-consuming process which delays response execution. Importantly though, this scenario only makes sense under the assumption that response execution is waiting for this additional process to transmit its output. Otherwise, based on stimulus-based response selection alone, the response threshold would be reached at exactly the same time for DO as for CO conditions. A parsimonious explanation for this additional “waiting time” could be that the response threshold is elevated under DO conditions because the “system” resides in a more controlled “goal-directed” mode under DO conditions (cf., Botvinick et al., 2001).

In conclusion, we speculate that effect anticipation plays an integral part in action planning even when it could solely rely on the antecedent stimulus. Importantly, this may be especially true early in practice, that is, before habitualization kicks in. Consistent with this view, relative response slowing under these circumstances indicates that effect-based action planning is a non-automatic process that may be different from the mechanisms that mediate the influence of effect representations after intensive practice. Furthermore, it will be especially important for future research to decide more clearly whether learning-related response slowing under DO conditions reflects either (S-)R-E learning itself or rather the active usage of these newly formed associations, as we would tentatively propose.

## Conflict of Interest Statement

The authors declare that the research was conducted in the absence of any commercial or financial relationships that could be construed as a potential conflict of interest.

## Footnotes

^1^When using SRep 1 as reference for online R-E learning progress (SRep1 – SRep2, Srep1 – SRep3, etc…), there was an overall trend for negative correlations with the RE compatibility effect but none of them reached significance (neither for RTs nor for errors).

^2^This holds under the assumption that factor Y and factor X impact RT along different timelines – otherwise factor X impact on performance slope should have a similar overshadowing effect regarding factor Y impact on performance slope.

^3^As a side note, it might be of interest that both error rates as well as RTs reached similar asymptotic levels for Exp. 1 (trial-and-error learning) and Exp. 2 (instruction-based learning).

^4^As a side note, it should be stressed that we consistently observed passive effect priming effects (i.e., R-E compatibility effects) in both experiments. This replicates an earlier observation made with a different priming procedure (Wolfensteller and Ruge, 2011) and provides further evidence that (i) R-E association can be rapidly learned when action selection takes place in a stimulus-based mode and (ii) the successful acquisition of R-E associations can be detected via the effect-priming rationale when test phase actions are selected in a stimulus-based action mode. A possible reason for different findings (Herwig et al., 2007; Pfister et al., 2011) might be that we investigate (S-)R-E learning in a very early phase of practice, hence precluding strong habitualization effects.

^5^See Discussion of Exp. 1 for an account that would assume amplified (S-)R-E learning due to increased unspecific DO-related distraction.
